# Overexpression of a Domain of Unknown Function 266-containing protein results in high cellulose content, reduced recalcitrance, and enhanced plant growth in the bioenergy crop *Populus*

**DOI:** 10.1186/s13068-017-0760-x

**Published:** 2017-03-23

**Authors:** Yongil Yang, Chang Geun Yoo, Hao-Bo Guo, William Rottmann, Kimberly A. Winkeler, Cassandra M. Collins, Lee E. Gunter, Sara S. Jawdy, Xiaohan Yang, Hong Guo, Yunqiao Pu, Arthur J. Ragauskas, Gerald A. Tuskan, Jin-Gui Chen

**Affiliations:** 1 BioEnergy Science Center and Biosciences Division, Oak Ridge National Laboratory, Oak Ridge, TN 37831 USA; 2 UT-ORNL Joint Institute for Biological Science, Oak Ridge National Laboratory, Oak Ridge, TN 37831 USA; 3 Department of Biochemistry & Cellular & Molecular Biology, University of Tennessee, Knoxville, TN 37996 USA; 4 ArborGen Inc., Ridgeville, SC 29472 USA; 5 Department of Chemical and Biomolecular Engineering & Department of Forestry, Wildlife, and Fisheries, University of Tennessee, Knoxville, TN 37996 USA

**Keywords:** Biofuel, Biomass, Cell wall, Cellulose, DUF266, *Populus*, Recalcitrance, Sugar release

## Abstract

**Background:**

Domain of Unknown Function 266 (DUF266) is a plant-specific domain. DUF266-containing proteins (DUF266 proteins) have been categorized as ‘not classified glycosyltransferases (GTnc)’ due to amino acid similarity with GTs. However, little is known about the function of DUF266 proteins.

**Results:**

Phylogenetic analysis revealed that DUF266 proteins are only present in the land plants including moss and lycophyte. We report the functional characterization of one member of DUF266 proteins in *Populus,* PdDUF266A. *PdDUF266A* was ubiquitously expressed with high abundance in the xylem. In *Populus* transgenic plants overexpressing *PdDUF266A* (*OXPdDUF266A*), the glucose and cellulose contents were significantly higher, while the lignin content was lower than that in the wild type. Degree of polymerization of cellulose in *OXPdDUF266A* transgenic plants was also higher, whereas cellulose crystallinity index remained unchanged. Gene expression analysis indicated that cellulose biosynthesis-related genes such as *CESA* and *SUSY* were upregulated in mature leaf and xylem of *OXPdDUF266A* transgenic plants. Moreover, *PdDUF266A* overexpression resulted in an increase of biomass production. Their glucose contents and biomass phenotypes were further validated via heterologous expression of *PdDUF266A* in Arabidopsis. Results from saccharification treatment demonstrated that the rate of sugar release was increased by approximately 38% in the *OXPdDUF266A* transgenic plants.

**Conclusions:**

These results suggest that the overexpression of *PdDUF266A* can increase cellulose content, reduce recalcitrance, and enhance biomass production, and that *PdDUF266A* is a promising target for genetic manipulation for biofuel production.

**Electronic supplementary material:**

The online version of this article (doi:10.1186/s13068-017-0760-x) contains supplementary material, which is available to authorized users.

## Background

The structural polysaccharides such as cellulose, hemicellulose, lignin, and pectin are important components of plant’s cell walls. Over 2000 genes have been estimated to be required for polysaccharide biosynthesis, assembly, and structural maintenance [1, 2]. Glycosyltransferases (GTs) are regarded as an important family of proteins participating in the synthesis of polysaccharides by transferring sugar moieties from an activated nucleotide sugar to a specific acceptor molecule [3]. Based on the amino acid sequence similarity, GT has been classified into 101 families designated as GT1 to GT101. These classifications are available in the carbohydrate-active enzyme (CAZy) database (http://www.cazy.org/GlycosylTransferases.html) [4]. Pectin, hemicellulose, and cellulose have been shown to be synthesized by at least one GT member as summarized in three recent review articles [5–7]. For example, galacturonosyltransferase (GAUT) 1, 7, and 8 that were classified as GT8 group members synthesize homogalacturonan of one pectin type among three different pectin polysaccharide types [8]. Other pectin polysaccharide types of Rhamnogalacturonan I and II are synthesized by arabinosyltransferase (ARAD) of GT47 [9, 10], galactosyltransferase (GAL) of GT92 [11], and xylosyltransferase of rhamnogalacturonan II (RGXT) of GT77 group [12, 13]. Xylogalacturonan synthesis, the third pectin polysaccharide type, is mediated by xylosyltransferase (XGD1) [14] classified in GT47-C. In general, pectin biosynthetic enzymes are localized to the Golgi apparatus and have been predicted as type-II transmembrane protein [5]. Hemicellulose biosynthesis is regulated by CELLULOSE SYNTHASE-LIKE PROTEIN (CSL), IRREGULAR XYLEM 7 and 8 (IRX7 and 8), and MUTANTS OF *ARABIDOPSIS THALIANA* WITH ALTERED CELL WALL POLYSACCHARIDE COMPOSITION (MUR) [6, 15].

About 1.7% of Arabidopsis annotated genes are predicted as GTs [15–17]. The functions of a large number of GTs still remain elusive. Domain of Unknown Function 266 (DUF266)-containing proteins (DUF266 proteins) share amino acid similarity with GT14 proteins, but Pfam database annotates DUF266 as a plant-specific domain and predicts them as ‘likely to be GT related’. An Arabidopsis GT14 member (AtGlcAT14A) was shown to function as β-glucuronosyltransferase involved in type-II arabinogalactan synthesis [18]. In Arabidopsis, a total of 14 DUF266 proteins (AtDUF266) were initially identified as being distantly related to GT14 group family [19]. Later on, Ye et al. identified a total of 22 AtDUF266 proteins by phylogenetic analysis of full-length amino acid sequences [20]. Twenty-seven *Populus* DUF266 proteins (based on *P. trichocarpa* annotation v2.0) were classified as GT14-LIKE proteins in this phylogenetic analysis [20]. Again, these DUF266/GT14-LIKE proteins formed a cluster that was phylogenetically distinct from the GT14 family members [20]. Subsequently, Lao et al. categorized AtDUF266 proteins as ‘not classified GT (GTnc)’ [21], to better reflect the uncharacterized features of this protein subfamily. So far, the only characterized DUF266 protein is rice BRITTLE CULM 10 (OsBC10) which has amino acid similarity with 2 β-1,6- *N*-acetylgalactosyltransferase (C2GnT) in animals [22]. In vitro enzymatic assay using Chinese hamster ovary cells revealed that OsBC10 has galactosyltransferase activity that is only ~1% of animal C2GnT [22]. Rice natural variants of *OsBC10* displayed phenotypic abnormalities such as small size of plant body and tiller number and brittleness of plant body. Glucose content was decreased in *Osbc10* mutant, and xylose, arabinose, and lignin contents were increased, indicating that OsBC10 influences cell wall composition [22]. OsBC10 was predicted to be a type-II intercellular membrane-binding protein and was shown to be localized in the Golgi complex [16, 22].

Except OsBC10, no other DUF266 proteins have been functionally characterized. In this study, we report the characterization of one member of *Populus* DUF266 protein (PdDUF266A). We provide evidence that PdDUF266A affects cellulose biosynthesis.

## Methods

### Amino acid sequence and phylogenetic analyses of DUF266 proteins

To identify DUF266 proteins in *Populus*, the full-length amino acid sequence of an Arabidopsis DUF266 protein, AT1G62305, was subjected to protein homolog search integrated in the Phytozome v11.0 (https://phytozome.jgi.doe.gov) [23]. Each identified PtDUF266 homolog was then used as a new query to search for DUF266 proteins in moss *(Physcomitrella patens*), lycophyte (*Selaginella moellendorffii*), rice (*Oryza sativa*), corn (*Zea mays*), soybean (*Glycine max*), *Amborella* (*Amborella trichopoda*), grape (*Vitis vinifera*), Arabidopsis (*Arabidopsis thaliana*), and Eucalyptus (*Eucalyptus grandis*) genomes. The identified DUF266 proteins from each species were subsequently used to perform reciprocal homolog search till no new DUF266 protein could be identified. The full-length amino acid sequences with >30% similarity (*e* value <0.01) to each input protein were selected and subjected to Pfam database [24] to validate the presence of the core-2/I branching domain, a hallmark of DUF266 proteins, and other possible motifs [16, 20]. The transmembrane domain (TM) was predicted by means of web-based TMHMM v2.0 (www.cbs.dtu.dk/servies/TMHMM) [25]. A probability value of 0.8 was used as a criterion to determine the presence of TM. Three-dimensional structure prediction was performed by means of the I-TASSER (iterative threading assembly refinement, v4.4) toolkit [26], and molecular recognition feature (MoRF) analysis of PdDUF266A was performed by means of the ANCHOR software with full-length amino acid sequence [27].

To conduct phylogenetic analysis, maximum likelihood (ML) tree was constructed with full-length amino acid sequences of collected DUF266 proteins. Compiled DUF266 proteins were aligned together by means of the MUSCLE software [28] integrated in the Geneious software (v8.1.2; Biomatters Ltd., New Zealand) with 12 maximum number of iterations together with kmer6_6 of distance measurement protocol under neighborhood joining clustering method. The best fitting model to construct maximum likelihood was calculated by ML option integrated in the MEGA 7 software [29]. The phylogenetic tree was constructed by selecting the model with the lowest value of Akaike Information Criterion (AIC) and Bayesian Information Criterion (BIC), and Maximum Likelihood values (InL). aLRT SH-like branch support method was used to improve likelihoods of branch and node.

### Plant materials

The full-length open-reading frame of *PdDUF266A* was amplified from *Populus deltoides* WV94. The complementary DNA (cDNA) was cloned into the pAGW560 binary vector in which the expression of *PdDUF266A* was driven by the *UBIQUITIN 3* promoter [30]. Agrobacterium-mediated transformation into *P. deltoides* genotype WV94 was conducted at ArborGen Inc. (Ridgeville, SC) as described previously [30]. These plants were transferred and grown in the greenhouse at Oak Ridge National Laboratory (Oak Ridge, TN) under constant 25 °C and 16 h/8 h photoperiod. To estimate stem cylinder volume, we measured plant height and stem base diameter of 6-month-old plants. We measured primary stem length from stem base (6 cm above soil surface) to shoot tip for plant height and measured the diameter of stem base using calipers.

### RT-PCR and quantitative RT-PCR analyses

To perform quantitative analysis of *PdDUF266A* transcript in the *Populus* transgenic plants, total RNA was extracted from the petiole of mature leaf of 6-month-old plants grown in the greenhouse by means of Sigma spectrum plant RNA extraction kit with modified Cetyltrimethyl Ammonium Bromide (CTAB) extraction buffer (Sigma-Aldrich, St. Louis, MO). One µg of total RNA was used to generate cDNA by means of the Rite aid reverse transcriptase following manufacturer’s instruction (Thermo Fisher Scientific, Hudson, NH). DreamTaq enzyme solution mixture (Thermo Fisher Scientific) was used for PCR reaction together with 1 µl of 2× diluted cDNA and gene-specific primers (Additional file 1). Gene-specific primers were designed from nonconserved DNA sequence region based on ClustalW DNA sequence alignment of *PdDUF266A* and its paralogs including Potri.002G227000, Potri.001G34840y, and Potri.015G045500 [31]. PCR reaction was performed as follows: denaturation at 95 °C for 2 min followed by 35 cycle of 95 °C for 30 s, 56 °C for 30 s, and 72 °C for 20 s. Another step of 72 °C for 7 min was performed for final extension reaction. Amplification of *Populus UBIQUITIN C* gene (*PdUBCc*, Potri.006G205700) was used as a control by the same PCR reaction but replacing annealing stage with 57 °C and cycle number of 25. The PCR product was run on 1% agarose gel with TBE (45 mM Tris–borate, 1 mM EDTA) at 100 V for 30 min. Gel image was taken by ChemiDoc XRS+ software (BIO-RAD, Hercules, CA).

For the expression pattern analysis of *PdDUF266A* in different tissues and organs, samples were collected between 12:00 hours and 2:00 p.m. from three WV94 plants (*Populus deltoides*). Total RNA were extracted from root, young leaf, mature leaf, young stem (internodes 1–3), mature stem (internodes 6–8), petiole of mature leaf, phloem (bark of mature stem) and xylem (scrapped stem under bark of mature stem) [32] by the same method as described above.

For quantitative RT-PCR (qRT-PCR) analyses, PCR reaction was performed using Maxima SYBR Green/ROX qPCR master mix including uracyl DNA glycosylase (UDG) (Thermo Fisher Scientific). 1 µl of 4× diluted cDNA was mixed together with gene-specific primers (Additional file 1). The PCR reaction was started with UDG activation at 50 °C for 2 min, a pre-denaturation of 95 °C for 10 min, and then followed by 40 cycles of combined two steps of 95 °C for 15 s and 60 °C for 30 s. The relative gene expression was calculated by 2 ^−ΔΔ*Ct*^ equation [33].

### Cell wall chemical composition analysis

The dried stem of 6-month-old *Populus* transgenic and wild type (WV94) plants were used for cell wall chemical composition analysis. The size of stem samples was reduced to 40 mesh by Wiley-mill (Thomas Scientific, Swedesboro, NJ) and Soxhlet-extracted with ethanol/toluene (1:2, v/v) for 24 h. The extractive-free sample was analyzed by the method consisting of two-step sulfuric acid (H_2_SO_4_) hydrolysis [34]. In the first step, the extractive-free sample was hydrolyzed with 72% (w/w) H_2_SO_4_ at 30 °C for 1 h. In the second step, the hydrolyzed sample was diluted to 4% H_2_SO_4_ (w/w) of final concentration, followed by autoclaving at 121 °C for 1 h. The hydrolysate was filtered from solid residue. The filtered liquid fraction was subjected to Dionex ICS-3000 ion chromatography system (Thermo Fisher Scientific, Sunnyvale, CA) for quantifying sugar contents. Total lignin content was quantified with acid-soluble and -insoluble lignin separation from hydrolysate and solid residue, respectively. Acid-soluble lignin was measured with liquid fraction at 240 nm wave length using UV/Vis spectroscopy. Acid-insoluble lignin was quantified with the filtered solid residue as described in the NREL procedure [34]. All analyses were technically duplicated from two different plants of the same transgenic line for statistical analysis. Cell wall compositional analysis with Arabidopsis transgenic plants expressing *PdDUF266A* was also conducted by the aforementioned method but without ethanol/toluene extraction.

### Cellulose content measurement using Anthrone assay

The mature stem tissue (internodes 6–9) of 6-month-old *Populus* transgenic and WV94 plants grown in greenhouse were dried and milled. A total of 15 mg of milled sample were dissolved in 500 µl of acetic nitric acid reagent [1: 8: 2 (v/v) of nitric acid: acetic acid: water] (Sigma-Aldrich) to measure the cellulose content according to the Updegraff’s method [35]. Heating was followed at 98 °C for 30 min. Insoluble fraction was pelleted by centrifugation for 10 min at 14,000 rpm. 600 μl of 67% sulfuric acid was added to the pellet followed by 1 h incubation at room temperature. Another centrifugation was performed for 5 min at 14,000 rpm to separate the solvent phase. 180 µl of deionized water was added to 20 µl solvent phase. Then 5× dilution was conducted. The freshly prepared anthrone solution (0.5 mg of anthrone/ml of concentrated sulfuric acid; Sigma-Aldrich) was mixed with the diluted solution. The mixture was boiled at 96 °C for 10 min followed by cooling down immediately at 4 °C. The absorbance was measured at 630 nm wave length by SpectraMax Plus 384 microplate reader (Molecular devices, Sunnyvale, CA). The glucose content was determined by means of the glucose standard curve [35]. The cellulose content percentage was calculated by applying the glucose content to the equation of [(Glucose quantity × 600 (dilution factor))/[15(initial sample amount) × 1000]] × 100. All analyses were technically repeated three times with two different plants of the same transgenic line.

### Degree of polymerization and crystallinity of cellulose using GPC and CP/MAS NMR

Degree of polymerization of cellulose from mature stem (internodes 6–9) of 6-month-old transgenic *Populus* was analyzed using Gel Permeation Chromatography (GPC) (SECurity GPC 1200 System, Polymer Standards Services, Warwick, RI) with four Waters Styragel columns (HR1, HR2, HR4, and HR6) and an Agilent UV detector [36]. Extractive-free biomass was delignified by peracetic acid (4 g peracetic acid/g biomass) at 25 °C for 24 h with stirring. The residual solid (holocellulose) was filtered, washed with DI water, and air-dried. The holocellulose was under consecutive extractions at room temperature with sodium hydroxide (NaOH; 17.5% for 2 h and 8.75% for additional 2 h) and washed with 1% (w/w) acetic acid and DI water for removing hemicellulose fraction. The cellulose (solid residues) fraction was freeze-dried and derivatized by tricarbanilation using anhydrous pyridine and phenyl isocyanate at 70 °C for 3 days, and then regenerated in methanol/water (7:3, v/v). The derivatized cellulose was vacuum-dried and dissolved in THF for GPC analysis. Molecular weight was calculated by Polymer Standards Service WinGPC Unity software with 12 polystyrene standards (1.2 × 10^3^ to 3.6 × 10^6^ g/mol). The weight-average degree of polymerization (DPw) of cellulose was calculated with the weight-average molecular weight (Mw) of the cellulose and the tricarbanilated cellulose unit (519 g/mol).

Crystallinity of cellulose was measured by means of cross polarization/magic angle spinning (CP/MAS) nuclear magnetic resonance (NMR) spectroscopy [37]. To determine the crystallinity of cellulose, cellulose was isolated from the holocellulose by HCl (2.5 M) hydrolysis at 100 °C for 1.5 h. The isolated cellulose was washed with DI water, and it maintained its moisture content at 30–50% prior to the NMR analysis. The NMR samples were packed into 4-mm cylindrical Zirconia MAS rotors and analyzed using a Bruker Avance III 400 MHz spectrometer operating at frequencies of 100.59 MHz for ^13^C in a Bruker double-resonance MAS probe head at spinning speeds of 8 kHz. CP/MAS experiments were carried out utilizing a 5 μs (90°) proton pulse, 1.5 ms contact pulse, 4 s recycle delay and 2048 scans. Each analysis was technically repeated three times with two different plants of each transgenic line.

### Sugar release assay

Dried and Wiley-milled (40 mesh) mature stems (internodes 6–9) of 6-month-old *Populus* transgenic and WV94 plants were used for sugar release measurement. About 250 mg of *Populus* sample (oven-dry weight) was loaded in 50 mM citrate buffer solution (pH 4.8) with Novozymes CTec2 (70 mg protein per gram of biomass; Franklinton, NC). The enzymatic hydrolysis was carried out at 50 °C with 200 rpm in an incubator shaker. Liquid hydrolysate was periodically collected at 0, 6, 12, 24, 48, and 72 h, and enzymes in the hydrolysate were deactivated in the boiling water before carbohydrates analysis. Released sugars in each hydrolysate were measured using Dionex ICS-3000 ion chromatography system. Each analysis was conducted in three technical replicates from single plant of each transgenic line.

### Statistical analysis

To determine statistical significance, *t* test of paired samples (against WV94) was performed at *p* < 0.01 by *t* test function integrated in Excel software (Microsoft, Redmond, WA). Asterisk in each figure indicates significant difference compared to WV94 (*p* < 0.01).

### Arabidopsis transgenic plants expressing *PdDUF266A*

The full-length open-reading frame of *PdDUF266A* was amplified from cDNA generated from WV94 petiole tissue by means of Phusion DNA polymerase (Thermo Fisher Scientific) with gene-specific primers (Additional file 1) according to manufacturer’s protocol. cDNA was generated as described in the RT-PCR and quantitative RT-PCR analyses section. The amplified PCR product was cloned into pENTR-TOPO/D (Invitrogen, Carlsbad, CA) followed by recombination into pGWB401 binary vector [38]. Transformation of *pGWB401::PdDUF266A* into Agrobacterium GV3101 was performed by means of the freeze–thaw method [39], and floral-dipping method was used for Arabidopsis (Col-0 ecotype) transformation [40]. The transformed Arabidopsis seedlings were screened on ½ Murashige and Skoog media (MS) (Plantmedia, Dublin, OH) medium plate supplemented with 1× Gamborg’s B-5 vitamin and 25 mg/l Kanamycin. For phenotypic analysis, Col-0 Arabidopsis was germinated on ½ MS medium without Kanamycin. Arabidopsis seedlings were transferred into soil and grown under 14 h/10 h photoperiod at 23 °C. To confirm heterologous expression of *PdDUF266A*, total RNA was extracted from rosette leaves of 4-week-old Arabidopsis plants using the same method as for RNA extraction from *Populus* samples described above. cDNA synthesis, RT-PCR reaction, and gel imaging were also performed by the same procedures as described above. Arabidopsis *ACTIN2* (*AtACT2*) was used for an internal control. PCR reaction was performed as follows: denaturation at 95 °C for 2 min followed by 30 cycle of 95 °C for 30 s, 56 °C for 30 s, and 72 °C for 20 s. Final extension at 72 °C for 10 min was used.

## Results

### DUF266 proteins and phylogenetic analysis

DUF266 proteins have only been reported in the plant kingdom. However, little is known about their functions, and the evolutional relationship of this protein family in different plant species remains elusive. In order to explore the function of DUF266 proteins, it was necessary to start with bioinformatics analysis. We identified DUF266 proteins from 10 plant species including moss (*P. patens*) and lycophyte (*S. moellendorffii*) representing ancient nonvascular- and nonseed-baring land plants, respectively; rice (*O. sativa*) and corn (*Z. mays*) of monocot, *Amborella* (*A. thrichopoda*) of gymnosperm woody plant; grape (*V. vinifera*), *Eucalyptus* (*E. grandis*), and *Populus* (*P. trichocarpa*) of angiosperm woody plant; and Arabidopsis (*A. thaliana*) and soybean (*G. max*) of dicot angiosperm herbaceous plants, respectively, by searching for proteins with amino acid similarity with AT1G62305, a previously reported DUF266 protein [19–21].

All identified DUF266 proteins had conserved core-2/I-branching domain which was designated as PF02485 domain in Pfam [24]. A total of 187 DUF266 proteins were identified from 10 plant genomes (Additional file 2, Additional file 3). The average amino acid length was 378 but the number of DUF266 proteins varied among plant species (Additional file 2, Additional file 3). Ye et al. identified 22 DUF266 proteins from Arabidopsis and 27 from *Populus* (based on *Populus trichocarpa* v2.0 genome annotation) [20]. Due to updated *Populus trichocarpa* genome annotation (v3.0), 25 *Populus* loci have been identified as DUF266 proteins in the present study. Current *Populus* genome (v3.0) no longer has four proteins previously reported by Ye et al. [20], and two newly annotated proteins were identified as DUF266 proteins (Potri.003G002400 and Potri.T037500) in the present study. Identified *Populus* DUF266 proteins are listed in the Additional file 4 with comparison of those identified by Ye et al. [20]. We also identified one additional rice DUF266 protein (Additional file 3) [22]. The majority of DUF266 proteins (~86% of all identified DUF266 proteins) possess a single TM at the N-terminus (Additional file 3). Besides TM and DUF266, no other functional domain was predicted in the protein domain analysis.

**Fig. 1:**
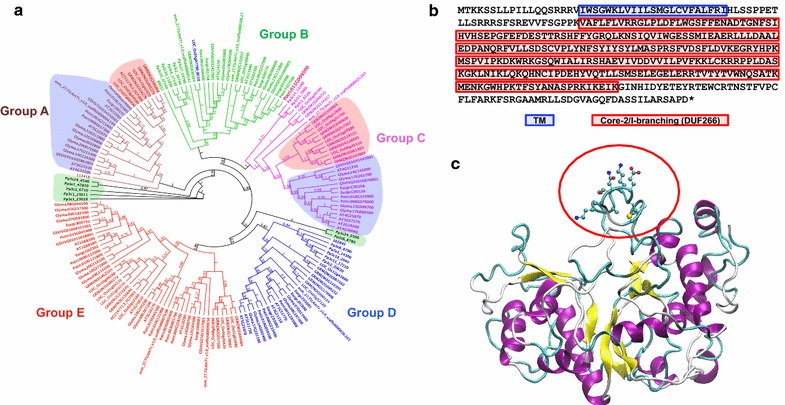
Bioinformatics analysis of DUF266 proteins. **a** Phylogenetic tree. A total of 169 DUF266 proteins were collected by amino acid sequence similarity analysis through Phytozome (v11.0) (https://phytozome.jgi.doe.gov/pz/portal.html). These 169 DUF266 proteins with 300–500 amino acids in length were identified from *Populus*, grape, *Eucalyptus*, soybean, Arabidopsis, rice, maize, *Amborella*, lycophyte, and moss. Shown is the maximum likelihood phylogenetic tree constructed by means of the mtREV model fitting method. aLRT SH-like branch support method was used to determine likelihoods of branch and node. PdDUF266A is marked in *red font*. Rice BC10 protein is indicated in *blue font*. Five groups (from A to E) are classified by clustering. The clades containing the monocot- (highlighted by *light red*), dicot- (highlighted by* light blue*) and moss-specific (highlighted by *light green*) DUF266 proteins are highlighted. **b** Amino acid sequence of PdDUF266A. The Core/I-branching/DUF266 domain and TM domain are indicated in *red* and *blue box*es, respectively. **c** Three-dimensional structure prediction and molecular recognition feature (MoRF) analysis of PdDUF266A by means of I-TASSER and ANCHOR. The MoRF region is composed of seven residues from A298 to K304 (ATKMENK) on PdDUF266A (marked by *red circle*). This region is predicted to have high potential to bind macromolecules

To examine the evolutional relationship of DUF266 proteins in different plant species, we performed maximum likelihood phylogenetic analysis. To improve the quality of alignment and phylogenetic tree construction, we excluded those DUF266 proteins with amino acid length <300 or >500 (Additional file 2; 18 DUF266 proteins). The selected 169 DUF266 proteins were subjected to amino acid sequence alignment and subsequently to the construction of maximum likelihood tree based on the alignment result (Additional file 5). As expected, the DUF266 domain is the only conserved domain among all aligned proteins and the N-terminal amino acid sequences were divergent (Additional file 5). Through the construction of maximum likelihood phylogenetic tree based on the MUSCLE alignment with filtered 169 DUF266 proteins, five clusters were distinctly formed, and subsequently these DUF266 proteins are designated as five different groups based on clustering (groups A to E) (Fig. 1a). Each cluster contains a set of DUF266 proteins from at least 8 different species except Group E which has no moss or lycophyte DUF266 proteins. All other four groups contain at least one moss DUF266 protein. All three lycophyte DUF266 proteins were associated in three different clades with close relationship with moss DUF266 proteins (Fig. 1a). Two clades contain only moss DUF266 proteins (Fig. 1a, highlighted in light green). In groups A and C, dicot and monocot nodes were clearly separated (Fig. 1a, dicot: highlighted in light blue; monocot: highlighted in light red).

### PdDUF266A protein structure prediction

No DUF266 protein has been characterized in *Populus*. In this study, we reported the functional characterization of one DUF266 protein in *Populus*, PdDUF266A (encoded by Potri.011G009500). PdDUF266A is 386 amino acids in length. It has a predicted TM at its N-terminus and the DUF266 at the C-terminus spanning ~73% of total amino acid sequences (Fig. 1b). A protein with indeterminate chromosomal location, Potri.T037500, was identified as having the highest amino acid sequence identity (89%) with PdDUF266A in *Populus*. No other predicted domains or motifs were previously reported in DUF266 proteins. We identified four conserved motifs within the DUF266 domain that share 50% amino acid identity with all examined DUF266 proteins (Additional file 6). Three-dimensional structure prediction by I-TASSER and accompanying MoRF analysis revealed a disorder fragment in PdDUF266A (Fig. 1c). A high potential binding motif of macromolecules such as nucleotide and metabolic chemical was identified from amino acid 298–304 on PdDUF266A (polypeptide of ATKMENK) and its paralog Potri.T037500 but not in other DUF266 proteins (Additional file 6).

### Expression pattern of *PdDUF266A*

**Fig. 2:**
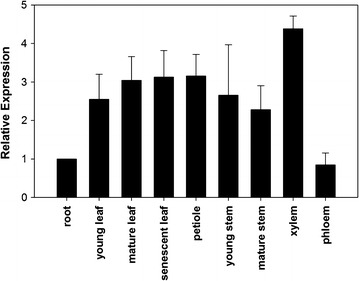
Expression analysis of *PdDUF266A*. qRT-PCR analysis of *PdDUF266A* expression across various tissues and organs including root, young leaf, mature leaf, young stem (internodes 1 to 3), mature stem (internodes 6 to 8), petiole of mature leaf, phloem (bark of mature stem), and xylem (scrapped stem under bark of mature stem). Relative expression was determined by comparing the *PdDUF266A* transcript level in other tissues and organs with that in root (set as 1). *PdUBCc* was used as an internal control. Shown are the mean values of three technical repeats ± S.D

As an attempt to investigate the function of *PdDUF266A*, we examined its gene expression patterns across various tissues and organs by qRT-PCR analysis with gene-specific primers. The transcript of *PdDUF266A* was detected in all tested tissues and organs. *PdDUF266*A transcript abundance was relatively high in xylem (scraping stem under bark of mature stem) and relatively low in phloem (bark of mature stem, Fig. 2). The result of qRT-PCR expression analysis is largely consistent with the RNAseq data in the *Populus* Gene Atlas study (Additional file 7).

### Cell wall chemical composition and expression of cellulose biosynthesis-related genes in *PdDUF266A* overexpression lines

**Fig. 3:**
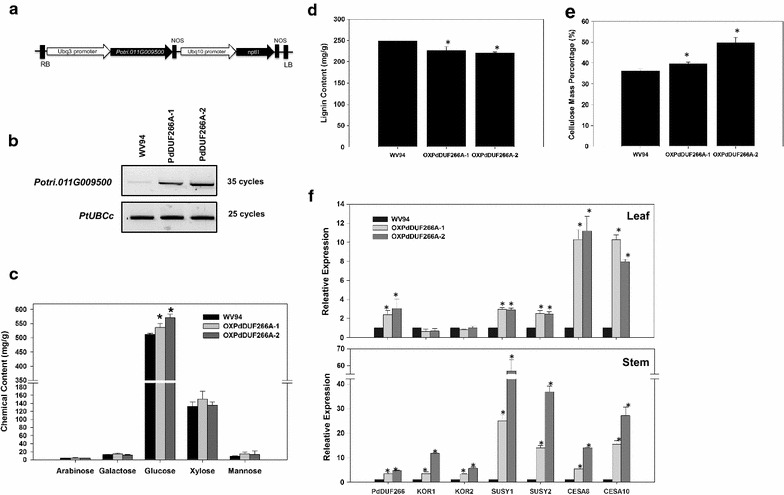
Cell wall composition and gene expression analyses in *OXPdDUF266A* transgenic lines. **a** Gene construct used for generating *Populus* transgenic plants overexpressing *PdDUF266A*. **b** RT-PCR analysis of *PdDUF266A* expression in two independent *Populus* transgenic lines overexpressing *PdDUF266A*. Total RNA was extracted from petiole of mature leaf. *PdUBCc* was used as an internal control. PCR cycle numbers are indicated. Transgenic lines (*OXPdDUF266A*-*1* and *OXPdDUF266A*-*2*) and WV94 (wild type) were grown under greenhouse conditions. **c** Sugar content analysis by means of ion chromatography after two-step acid treatment. **d** Total lignin content by measuring acid soluble/insoluble separation. **e** Cellulose content analysis by means of Anthrone dye staining. **f** qRT-PCR analysis of cellulose biosynthesis-related genes in mature leaf and xylem. *PdUBCc* was used as an internal control to normalize the qRT-PCR result. Shown are mean values of three technical repeats from two biological repeats for each transgenic line ± S.D. *Asterisks* indicate statistical significance (*p* < 0.01)

To characterize the function of *PdDUF266A*, *Populus* transgenic plants overexpressing *PdDUF266A* (*OXPdDUF266A*) were generated. Among eight transgenic lines, we selected two transgenic lines that had relatively high *PdDUF266A* gene expression (Additional file 8). The transcript level of *PdDUF266A* was examined again in two selected independent *Populus* transgenic lines (renamed *OXPdDUF266A*-*1* and *OXPdDUF266A*-*2* for simplicity) by RT-PCR with *PdDUF266A* gene-specific primers. Both *OXPdDUF266A* lines were confirmed to overexpress *PdDUF266A*. (Fig. 3b).

We investigated the carbohydrate composition in stem tissues by means of ion chromatography after two-step sulfuric acid hydrolysis procedures. *OXPdDUF266A*-*1* and *OXPdDUF266A*-*2* transgenic plants had significantly higher glucose contents (4.8 and 11.4% increases, compared to WV94, respectively) whereas had no significant alteration in the contents of arabinose, galactose, xylose or mannose (*p* < 0.01; Fig. 3c). Both transgenic lines had lower lignin content than WV94 (Fig. 3d). Therefore, we focused on the high glucose content phenotype in *OXPdDUF266A* transgenic lines to further investigate its impact on saccharification treatment.

To verify whether high glucose content observed in the *OXPdDUF266A* transgenic lines was due to high cellulose content, total cellulose content in stem was estimated by measuring glucose monomer with anthrone staining method. *OXPdDUF266A* lines had higher cellulose contents (7.6 and 37.1% increases, respectively) compared with that of WV94 (Fig. 3e). This observation supports that high glucose content identified by chemical composition analysis is mainly due to high cellulose content in *OXPdDUF266A* lines.

In order to seek further evidence to support an influence of *PdDUF266A* on cellulose biosynthesis, we conducted qRT-PCR analysis of the expression of representative cellulose biosynthesis-related genes such as *CELLULOSE SYNTHASE* (*CESA*), *SUCROSE SYNTHASE* (*SUSY*), and *KORRIGAN* (*KOR*) [7, 41]. *KOR* encodes a putative membrane bound β-1,4 endoglucanase [42]. Its expression was not altered in leaf tissue of *OXPdDUF266A* lines whereas the transcript levels of two *CESA* and two *SUSY* genes were significantly increased in the *OXPdDUF266A* lines (*p* < 0.01, Fig. 3f). In xylem, the transcript levels of *KOR*, *SUSY,* and *CESA* were all significantly increased (Fig. 3f). These expression analyses supported that PdDUF266A affects cellulose biosynthesis-related gene expression.

### Characteristics of cellulose and sugar releases

Through cell wall chemical characterization and gene expression analyses, we found that PdDUF266A potentially affects cellulose synthesis. We further examined the degree of polymerization of cellulose by gel permeation chromatography (GPC). The degree of polymerization of cellulose in *OXPdDUF266A* lines was higher than that in WV94. On average, cellulose in *OXPdDUF266A*-*1* and *OXPdDUF266A*-*2* contains 504 and 618 more glucose units (10.2 and 12.5% increases) than that in WV94, respectively (Table 1).

**Table 1:** Degree of polymerization of cellulose in *OXPdDUF266A* transgenic lines

Genotype	DPw^a^
WV94	4946 ± 30
*OXPdDUF266A*-*1*	5450 ± 4^b^
*OXPdDUF266A*-*2*	5564 ± 64^c^

The cellulose characterization was performed with milled stem samples by gel permeation chromatography. Each analysis was technically duplicated with two different plants of each transgenic line. Shown are mean values ± S.D. (*n* = 2)

^a^The weight-average degree of polymerization (DPw) = Mw/519

^b, c^Indicate statistical significances of *p* < 0.05 and *p* < 0.01, respectively, in comparison with WV94

Cellulose is the major cell wall component for biofuel production. Cellulose crystallinity plays an important role in enzymatic activity on cellulose hydrolysis [43, 44]. To examine whether the high cellulose content in the *OXPdDUF266A* lines may alter cellulose crystallinity, we estimated cellulose crystallinity index (CrI) of *OXPdDUF266A* lines by CP/MAS NMR. As shown in Table 2, no considerable alteration in CrI was observed between *OXPdDUF266A* lines and WV94, indicating that high cellulose content in *OXPdDUF266A* lines did not alter cellulose crystallinity.

**Table 2:** Cellulose crystallinity index in *OXPdDUF266A* transgenic lines

Genotype	Crystallinity (Crl)
WV94	0.52
*OXPdDUF266A*-*1*	0.53
*OXPdDUF266A*-*2*	0.52

The cellulose crystallinity was measured by the cross polarization magic angle spinning NMR analysis. Each analysis was technically repeated three times with two different plants of each transgenic line. Note that cellulose crystallinity was not significantly (*p* < 0.05) altered in *OXPdDUF266A* transgenic lines

**Fig. 4:**
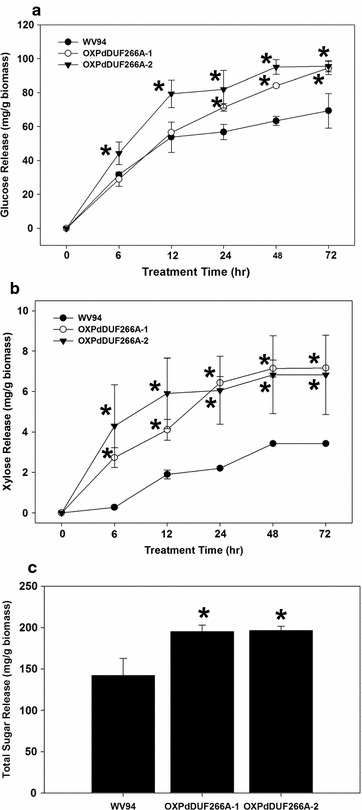
Saccharification efficiency of *OXPdDUF266A* transgenic plants. Dried *Populus* stem was used for this analysis. **a** Glucose release. **b** Xylose release. *X axis* denotes enzymatic hydrolysis times in (**a**) and (**b**). **c** Total sugar release at 72-h enzymatic hydrolysis. Shown are mean values of two biological replicates ± standard deviation. *Asterisks* indicate statistical significance (*p* < 0.01)

To assess the sugar release performance of the *OXPdDUF266A* lines, glucose and xylose releases during the enzymatic hydrolysis were monitored. At 6-h hydrolysis, the *OXPdDUF266A*-*2* line already had higher glucose release than WV94 (Fig. 4a). More xylose was also released from both *OXPdDUF266A* lines than WV94 at 6-h hydrolysis (Fig. 4b). At the final time point of 72-h enzymatic hydrolysis, the glucose amounts released from *OXPdDUF266A*-*1* and *OXPdDUF266A*-*2* were 36.2 and 37.9% higher than that from WV94, respectively (Fig. 4a), and the xylose released from the *OXPdDUF266A* line was over twofolds greater than that from WV94 (Fig. 4b). The total released sugar amounts at 72-h hydrolysis from *OXPdDUF266A*-*1* and *OXPdDUF266A*-*2* lines were increased by 37.3 and 38.2%, respectively, compared to that from WV94 plant (Fig. 4c). When the sugar release rate was calculated based on glucose and xylose contents, both *OXPdDUF266A* transgenic lines also had higher rates of glucose and xylose releases (Table 3). This result indicates that the increase of sugar release could be from both higher cellulose content and increased saccharification rate in the *OXPdDUF266A* lines.

**Table 3:** Sugar release yields based on glucose and xylose contents in plant cell walls

	Glucose (%)	Xylose (%)
WV94	13.5	2.6
*OXPdDUF266A*-*1*	16.6	4.8
*OXPdDUF266A*-*2*	17.8	5.0

Shown are sugar releases at 72 h enzymatic hydrolysis

### Overexpression of *PdDUF266A* increases biomass production

**Fig. 5:**
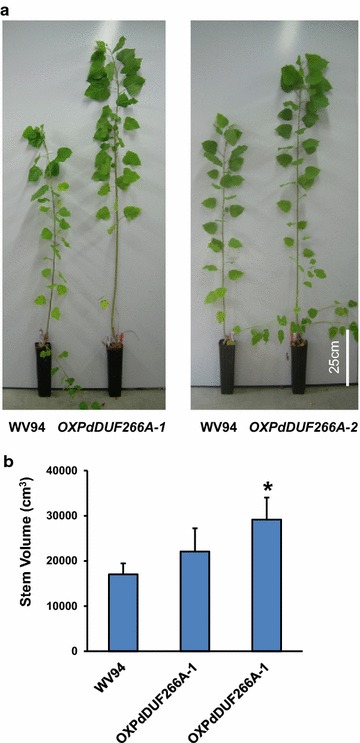
Biomass amounts of *OXPdDUF266A* transgenic plants. **a** 6-month-old *OXPdDUF266A* plants grown under greenhouse conditions. **b** Estimation of stem volume. Height and diameter were measured in each plant. The volume was estimated by means of the πr^2^h equation. Shown are mean values ± s.e. (*n* = 24, 5, and 3 for WV94, *OXPdDUF266A*-*1*, and *OXPdDUF266A*-*2*, respectively). *Asterisk* significant difference from WV94 (*p* < 0.01)

We noticed that *Populus* transgenic plants overexpressing *PdDUF266A* were constantly larger than control plants under greenhouse conditions. Therefore, we measured the diameter and height and used the stem volume to estimate the biomass amounts of *OXPdDUF266A* plants and compared them with the WV94 control plants. As shown in Fig. 5, the stem volumes of *OXPdDUF266A*-*1* and *OXPdDUF266A*-*2* plants were higher than that of the control plant, indicating that overexpression of *PdDUF266A* increases biomass production.

### Heterologous expression of *PdDUF266A* in Arabidopsis

**Fig. 6:**
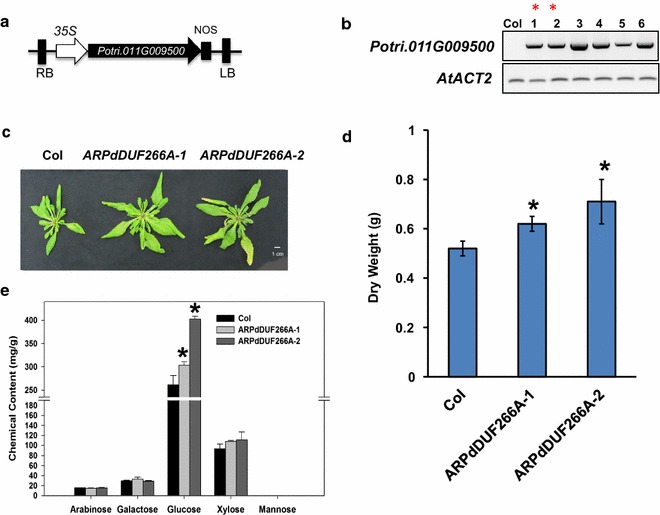
Characterization of Arabidopsis transgenic plants heterologously expressing *PdDUF266A*. **a** Gene construct used for generating Arabidopsis transgenic plants. **b** RT-PCR analysis of *PdDUF266A* in Arabidopsis transgenic lines. Two lines (marked with *asterisk*) were selected for further analysis. **c** Comparison of rosette size of 6-week-old Arabidopsis plants. **d** Dry weight of Arabidopsis transgenic plants. **e** Cell wall chemical compositional analysis of 6-week-old Arabidopsis plants. Shown in panels (**d**) and (**e**) are mean values of three biological replicates ± S.D. *Asterisk* indicate statistical significance (*p* < 0.01, *n* = 3)

As a way to further validate *PdDUF266A*’s function, we introduced *PdDUF266A* into Arabidopsis and generated Arabidopsis transgenic lines heterologously expressing *PdDUF266A*. We used the constitutive *35S* promoter to drive the expression of *PdDUF266A* in Arabidopsis and obtained several transgenic lines expressing *PdDUF266A* (Fig. 6a, b). Two independent Arabidopsis transgenic lines (*ARPdDUF266A*-*1* and *ARPdDUF266A*-*2*) were selected for further analysis. 6-week-old Arabidopsis plants were used for chemical composition analysis and dry weight measurement. The rosette size of both *ARPdDUF266A* lines was visibly larger than that of wild type (Fig. 6c). The dry weights of *ARPdDUF266A*-*1* and *ARPdDUF266A*-*2* were 17.1 and 34.1%, respectively, more than that of Col (Fig. 6d). The result of chemical compositional analysis of whole dried plant indicated that the glucose contents in *ARPdDUF266A*-*1* and *ARPdDUF266A*-*2* were increased by 16.0 and 53.9%, respectively, compared with Col (Fig. 6e). Other cell wall chemicals did not differ significantly between the transgenic lines and wild-type plants. Collectively, these results suggest that heterologous expression of *PdDUF266A* increases cellulose content and biomass production in Arabidopsis and support that *PdDUF266A* affects cellulose biosynthesis and biomass production in *Populus*.

## Discussion

### Bioinformatics of DUF266 proteins

As the Pfam database sorting protein families and functional domain was used for annotating protein sequences from sequenced genomes, most proteins have been categorized into different protein families based on the aligned protein domain. However, many protein domains have remained uncharacterized or poorly characterized, particularly the DUF. Currently, 3786 DUF protein families are present in the Pfam database [45]. In plants, DUF23, DUF246, and DUF266 were bioinformatically associated with GT families, but characterization of these proteins has been scant [16]. Among them, DUF266 proteins have been categorized as ‘not classified GT (GTnc)’ [21].

In this study, we identified DUF266 proteins in 10 plant species including moss, lycophyte, corn, rice, *Amborella*, *Populus*, grape, *Eucalyptus*, Arabidopsis, and soybean. Chlorophyte genome did not contain any DUF266 proteins when the protein similarity analysis was performed with moss DUF266 domain of Pp3c1_23610 or with the DUF266 domain of PdDUF266A (data not shown). A total of 187 DUF266 proteins were identified in these 10 different plant species (Additional files 2, 3). It appeared that DUF266 proteins are only present in embryophytes. Maximum likelihood phylogenetic tree clustered those proteins into five different clades (Fig. 1). The fewer number of GTs in lycophyte, *S. moellendroffii*, was also reported previously in the genome analysis [46]. Cell walls in *S. moellendroffii* display identical cell wall composition with higher plant species, but vascular formation differs [46]. This is consistent with the notion that *S. moellendroffii*, a lycophyte, has overlapped features between ancient and higher land plant species.

The amino acid sequence similarity analysis showed the most DUF266 proteins have conserved DUF266 domain at their C-terminus which covers approximately 70% of total amino acid sequences, whereas N-terminal sequences have poor amino acid similarity and vary in length. DUF266 domain also displays variations in amino acid sequence. For example, the disordered sequence was only identified in the DUF266 domain of PdDUF266A and its paralog (Additional file 6), implying that some members of DUF266 family proteins may have specialized functions.

### PdDUF266A and cellulose biosynthesis

Glycosyltransferase family proteins occupy a large portion of the enzyme group involved in polysaccharide and glycoprotein syntheses. CAZy database indicates that 450 Arabidopsis and 600 rice genes are included in GT family [21]. The subcellular localization analysis of a subset of Arabidopsis and rice GTs by bombarding the gene into yellow onion epidermal cells indicated that most GTs are localized in Golgi or endoplasmic reticulum (ER) [21]. Since DUF266 domain was recently merged into Core2/I-branching domain in Pfam database [24], DUF266 proteins are considered as homologs of 1-6-*N*-acetylglucosaminyltransferase and β-xylosyltransferase synthesizing *O*-glycans in animal [47, 48]. However, bioinformatics analysis indicated that DUF266 proteins were distantly related to GT14, implying that their protein activity is likely not identical to GT14 [16, 20]. To date, OsBC10, a rice DUF266 protein, is the only characterized DUF266 protein [22]. However, the C2GnT activity of OsBC10 was only ~1% of animal C2GnT [22], implying that the C2GnT activity may not represent the primary function of OsBC10. It should be noted that although DUF266 domain is derived from the Core-2/I-branching domain, DUF266 proteins are unique in plants, whereas Core-2/I-branching domain-containing proteins can be found in animals and bacteria.

Results from microarray data indicated that many *Populus DUF266* genes are expressed in the developing xylem, implying they may potentially function in cell wall biosynthesis [20]. As shown in Fig. 2, our expression analysis showed that *PdDUF266A* transcript was detected with relatively high abundance in xylem. This result is consistent with a role of *PdDUF266A* in secondary cell wall biosynthesis. In the chemical compositional analysis of stem tissues, *OXPdDUF266A* transgenic lines had higher glucose and cellulose contents but lower lignin content than that in wild type (Fig. 3). The high contents of glucose and cellulose in the *PdDUF266A* overexpression lines agrees with the opposite effect of the *Osbc10* mutant [22], implying that the influence of cellulose biosynthesis may be a common feature of DUF266 proteins.

The role of *PdDUF266A* in cellulose biosynthesis was further supported by the expression analysis of cellulose biosynthesis-related genes and heterologous expression in Arabidopsis. The expression of cellulose biosynthesis-related genes such as *KOR*, *CESA,* and *SUSY* were increased in *OXPdDUF266A* transgenic lines (Fig. 3). Arabidopsis transgenic plants heterologously expressing *PdDUF266A* had higher glucose content compared with wild type (Fig. 6). We also observed that the degree of polymerization of cellulose was higher in the *OXPdDUF266A* transgenic plants (Table 1) whereas cellulose crystallinity was not affected (Table 2), indicating that high cellulose content did not alter amorphous cellulose composition in *OXPdDUF266A* transgenic plants. However, how PdDUF266A affects cellulose biosynthesis, degree of polymerization, or the expression of cellulose biosynthesis-related gene remains unknown. The biochemical property of PdDUF266A is worth further investigation.

## Conclusions

Higher cellulose content, better sugar release performance, and enhanced biomass production are phenotypes of *OXPdDUF266A* transgenic plants identified in this study. Plant biomass has become an important source for biofuel production. Cellulose, hemicellulose, and lignin are all important targets for genetic modification for improving feedstock quality. Higher cellulose content (increased by 37.1%) and enhanced sugar release (increased by 38.2% of total sugar release) in *OXPdDUF266A* transgenic plants make *PdDUF266A* a promising target for genetic manipulation for improving biomass quality. Increase in biomass production observed in *OXPdDUF266A* transgenic plants makes *PdDUF266A* an even more attractive target in that both biomass quality and quantity can be simultaneously improved via overexpression.

## Additional files

**Additional file 1.** List of primers used for RT-PCR and qRT-PCR analyses in this study.

**Additional file 2.** Distribution of DUF266 proteins in 10 different species. Number denotes total number of DUF266 proteins identified from each species. Numbers in parenthesis indicated DUF266 proteins with <300 or >500 amino acids.

**Additional file 3.** List of DUF266 proteins in 10 different plant species. A total of 187 DUF266 proteins were identified from 10 plant species including moss (*P. patens*), lycophyte (*S. moellendorffii)*, rice (*O. sativa*), corn (*Z. mays*), soybean (*G. max*), *Amborella* (*A. thrichopoda*), grape (*V. vinifera*), *Eucalyptus* (*E. grandis*), *Populus* (*P. trichocarpa*) and Arabidopsis (*A. thaliana*).

**Additional file 4.** PtDUF266 family members in the present study (based on *Populus trichocarpa* genome annotation v3.3) and comparison with previously identified PtDUF266 proteins by Ye et al. [20] (based on *Populus trichocarpa* genome annotation v2.0).

**Additional file 5.** Amino acid alignment of DUF266 proteins from 10 different species by using MUSCLE. A total of 187 DUF266 proteins were identified from 10 plant species including moss (*P. patens*), lycophyte (*S. moellendorffii)*, rice (*O. sativa*), corn (*Z. mays*), soybean (*G. max*), *Amborella* (*A. thrichopoda*), grape (*V. vinifera*), *Eucalyptus* (*E. grandis*), *Populus* (*P. trichocarpa*) and Arabidopsis (*A. thaliana*). Shown is amino acid sequence alignment of 169 DUF266 proteins excluding those with <300aa or >500aa in length. The DUF266 domain is shown in blue box. Proteins with extra amino acid sequences or lacking N-terminal sequences are marked by arrows (Red arrows: with extra amino acids; blue arrows, lacking N-terminal sequences; green arrows, lacking C-terminal sequences within the DUF266 domain). Four corn DUF266 proteins having longer N-terminal sequences than others are indicated by purple arrows.

**Additional file 6.** Protein motif analysis of PdDUF266A. a) Conserved motifs identified in the DUF266 domain. Four conserved regions were identified with 50% amino acid identity threshold. The predicted disordered region is marked in a red box in the diagram. b) Amino acid alignment of ATKMENK residues. ATKMENK fragment is marked in blue box. PdDUF266A is indicated by a black arrow. Sequence logo displays conserved amino acid sequence with over 50% similarity.

**Additional file 7.** *Potri.011G009500* expression in different tissues and organs. Normalized FPKM values for various tissues and organ were compiled from *Populus* Gene Atlas dataset integrated in the Phytozyme website (https://phytozome.jgi.doe.gov).

**Additional file 8.** RT-PCR analysis of *PdDUF266A* transcript in *Populus* transgenic lines.

## Abbreviations

DUF266: Domain of Unknown Function 266
GPC: gel permeation chromatography
GT: glycosyltransferase
NMR: nuclear magnetic resonance
OsBC10: rice BRITTLE CULM 10
TM: transmembrane domain
