# Supplementary figures and images for: Overexpression of a Domain of Unknown Function 266-containing protein results in high cellulose content, reduced recalcitrance, and enhanced plant growth in the bioenergy crop Populus
Source: Biotechnol Biofuels. 2017 Mar 23;10:74. doi: 10.1186/s13068-017-0760-x (PMC5364563; doi:10.1186/s13068-017-0760-x)

## Slide 1
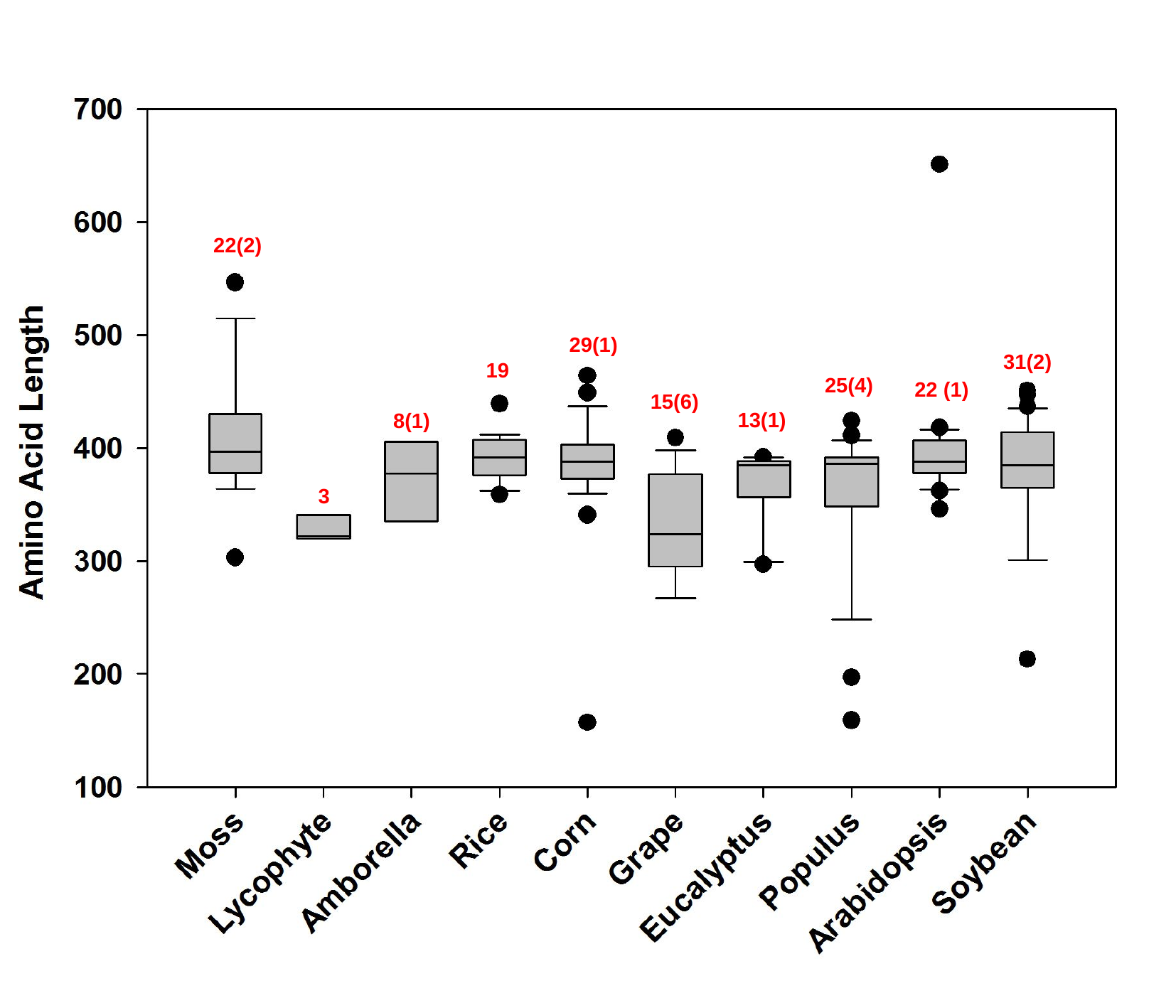

22(2)
29(1)
31(2)
19
25(4)
22 (1)
15(6)
13(1)
8(1)
3

Supplement: Supplementary file 2 — Additional file 2. Distribution of DUF266 proteins in 10 different species. Number denotes total number of DUF266 proteins identified from each species. Numbers in parenthesis indicated DUF266 proteins with <300 or >500 amino acids. [file 13068_2017_760_MOESM2_ESM.pptx]

## Slide 1
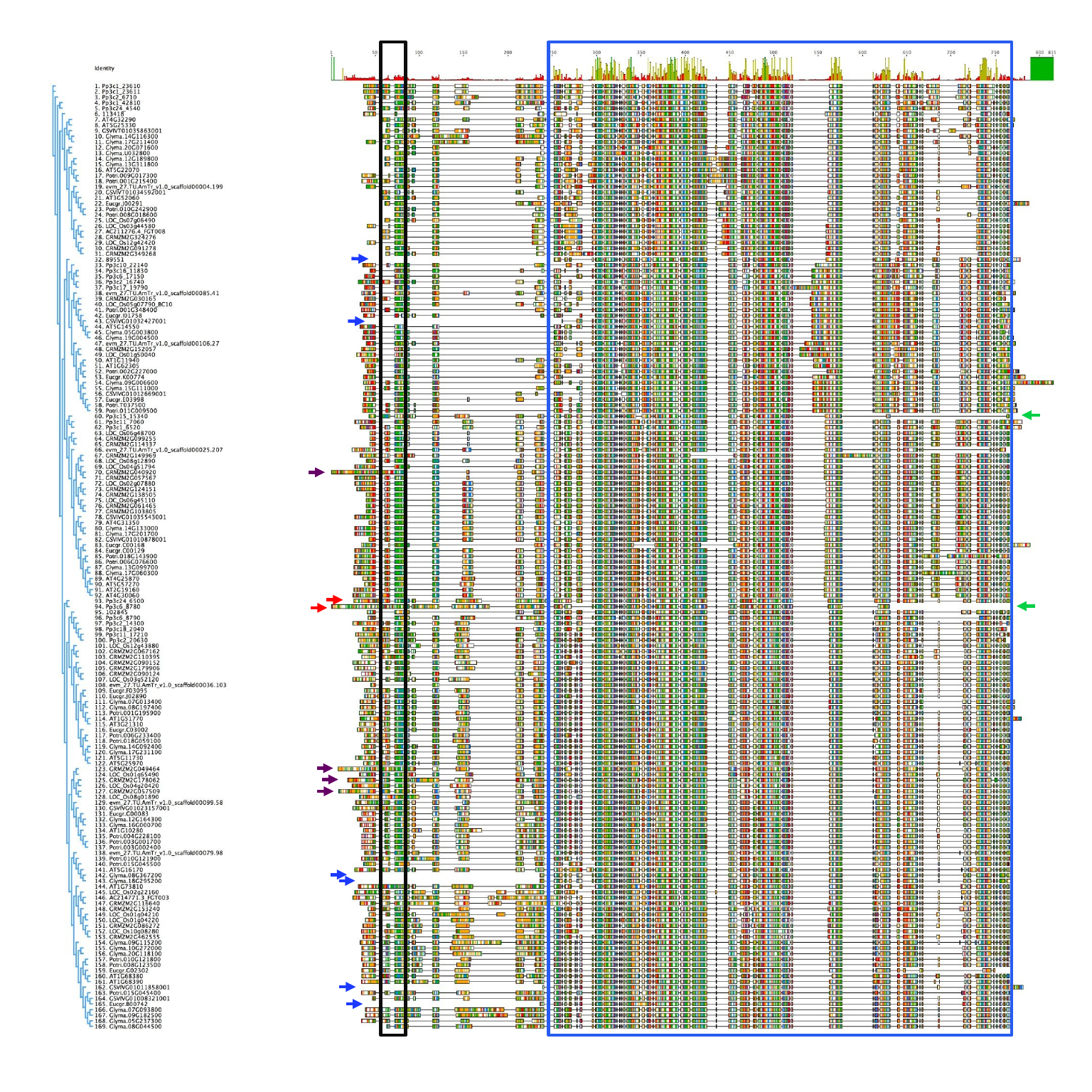

Supplement: Supplementary file 5 — Additional file 5. Amino acid alignment of DUF266 proteins from 10 different species by using MUSCLE. A total of 187 DUF266 proteins were identified from 10 plant species including moss (P. patens), lycophyte (S. moellendorffii), rice (O. sativa), corn (Z. mays), soybean (G. max), Amborella (A. thrichopoda), grape (V. vinifera), Eucalyptus (E. grandis), Populus (P. trichocarpa) and Arabidopsis (A. thaliana). Shown is amino acid sequence alignment of 169 DUF266 proteins excluding those with <300aa or >500aa in length. The DUF266 domain is shown in blue box. Proteins with extra amino acid sequences or lacking N-terminal sequences are marked by arrows (Red arrows: with extra amino acids; blue arrows, lacking N-terminal sequences; green arrows, lacking C-terminal sequences within the DUF266 domain). Four corn DUF266 proteins having longer N-terminal sequences than others are indicated by purple arrows. [file 13068_2017_760_MOESM5_ESM.pptx]

## Slide 1
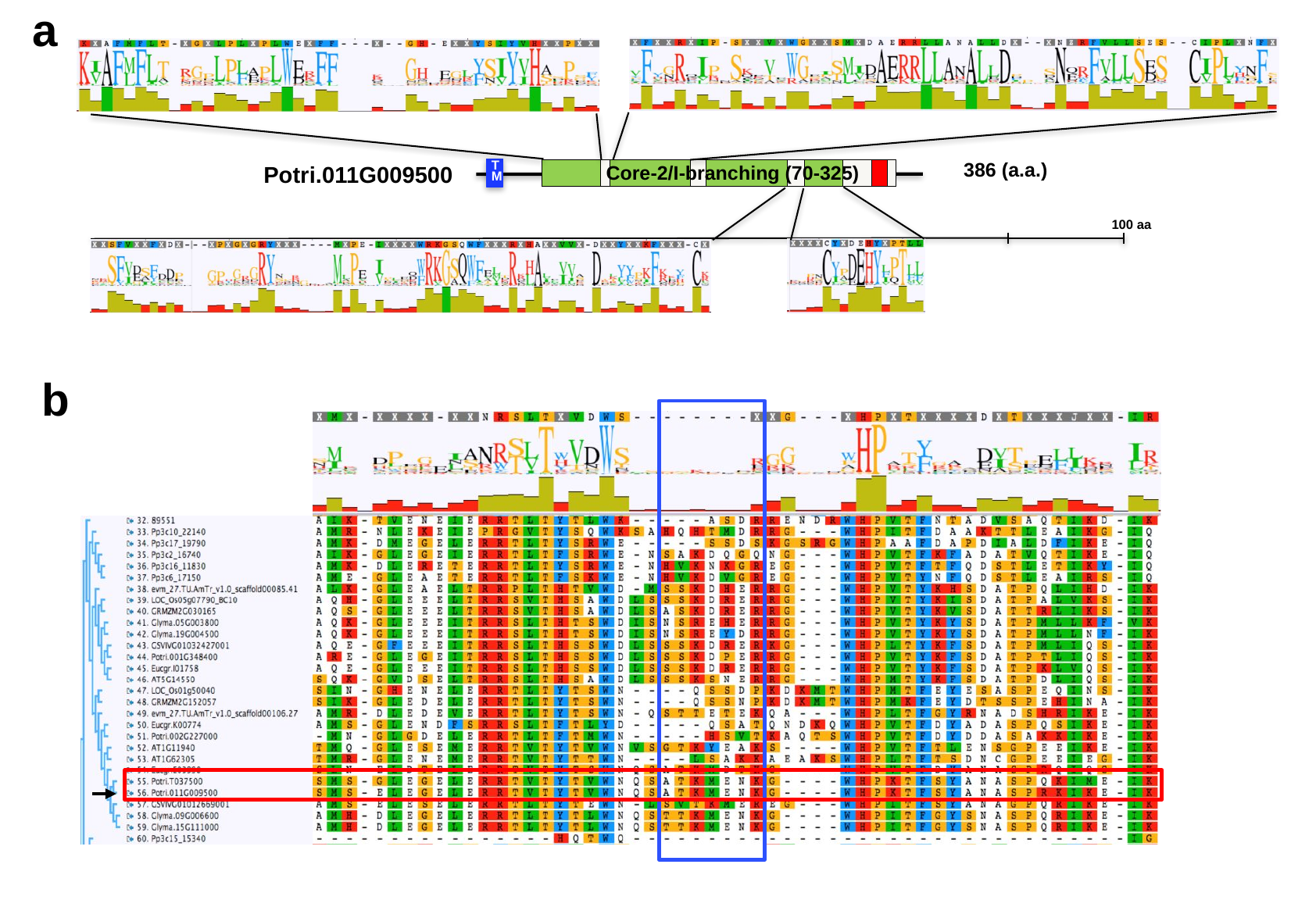

a
386 (a.a.)
Core-2/I-branching (70-325)
Potri.011G009500
TM
100 aa
b

Supplement: Supplementary file 6 — Additional file 6. Protein motif analysis of PdDUF266A. a) Conserved motifs identified in the DUF266 domain. Four conserved regions were identified with 50% amino acid identity threshold. The predicted disordered region is marked in a red box in the diagram. b) Amino acid alignment of ATKMENK residues. ATKMENK fragment is marked in blue box. PdDUF266A is indicated by a black arrow. Sequence logo displays conserved amino acid sequence with over 50% similarity. [file 13068_2017_760_MOESM6_ESM.pptx]

## Slide 1
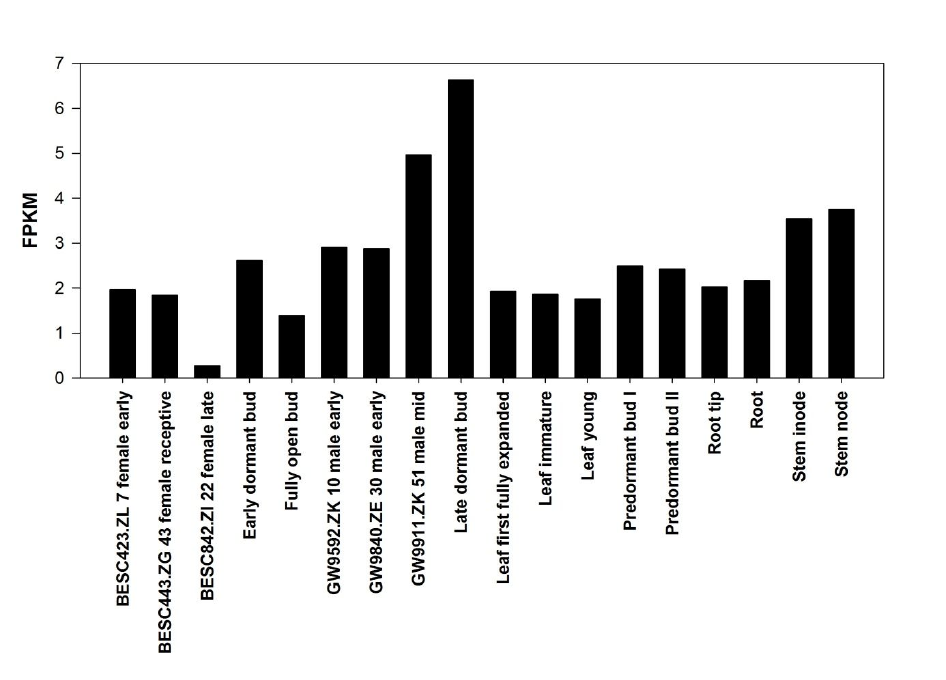

Supplement: Supplementary file 7 — Additional file 7. Potri.011G009500 expression in different tissues and organs. Normalized FPKM values for various tissues and organ were compiled from Populus Gene Atlas dataset integrated in the Phytozyme website (https://phytozome.jgi.doe.gov). [file 13068_2017_760_MOESM7_ESM.pptx]

## Slide 1
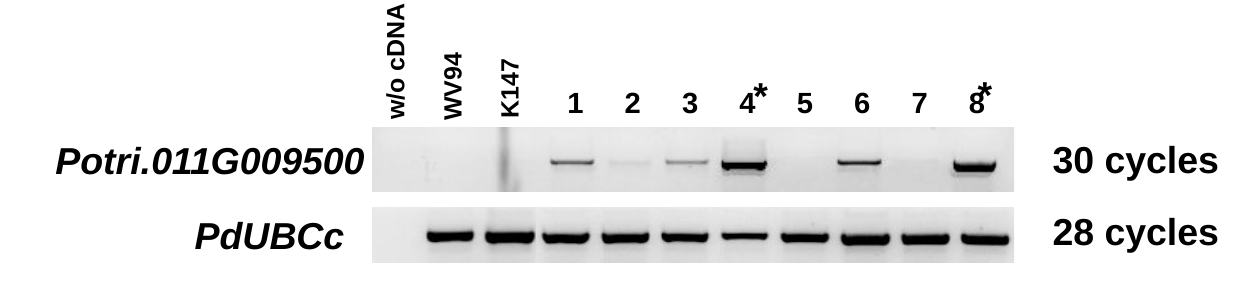

w/o cDNA
WV94
K147
*
*
 1 2 3 4 5 6 7 8
30 cycles
Potri.011G009500
28 cycles
PdUBCc

Supplement: Supplementary file 8 — Additional file 8. RT-PCR analysis of PdDUF266A transcript in Populus transgenic lines. [file 13068_2017_760_MOESM8_ESM.pptx]
